# Enhancing diagnostic logic in high-acuity care: evidence from an online flipped classroom intervention in emergency medicine

**DOI:** 10.1186/s12909-026-09094-x

**Published:** 2026-04-09

**Authors:** Ali Delirrooyfard, Mehdi Sayyah, Soleiman Ahmady, Noushin Kohan

**Affiliations:** 1 Department of Emergency Medicine, School of Medicine, Imam Khomeini Hospital, Ahvaz Jundishapur University of Medical Sciences, Ahvaz, Iran; 2 Education Development Center (EDC), Ahvaz Jundishapur University of Medical Sciences, Ahvaz, Iran; 3 School of Medical Education and Learning Technology, Shahid Beheshti University of Medical Sciences, Tehran, Iran; 4 Department of LIME, Research Affiliated Faculty, Karolinska Institute, Stockholm, Sweden; 5 Department of Medical Education, Department of eLearning in medical education, Smart University of Medical Sciences, Tehran, Iran

**Keywords:** Flipped Learning, Clinical Reasoning, Emergency Medicine, Education, Medical, Undergraduate, Educational Measurement, Computer-Assisted Instruction, Diagnostic Logic, Blended Learning

## Abstract

**Background:**

Medical education has increasingly embraced the flipped classroom—a pedagogical model involving pre-class online instruction followed by active problem-solving. However, its effectiveness in clinical settings where knowledge application is critical, such as clinical reasoning, requires further investigation. This study evaluated whether an online flipped classroom (OFC) approach enhances clinical reasoning performance among medical students during their emergency medicine clerkship.

**Methods:**

A quasi-experimental, single-group pre-test–post-test design was employed involving 45 medical students at Jundishapur University of Medical Sciences (2022–2023. A clinical reasoning course was developed based on a modified ADDIE instructional design model. The intervention included asynchronous e-content delivered via WhatsApp and a Learning Management System, followed by 60-minute synchronous bedside think-aloud sessions. Clinical reasoning was assessed using Objective Structured Clinical Examinations and the Clinical Reasoning Indicators History Taking Scale (CRI-HT-S).

**Results:**

A total of 45 medical students (40% male, 60% female) participated. Statistically significant differences were found between the total mean scores before and after the intervention. The total mean score increased from 25.97 ± 2.40 (average range) to 30.40 ± 3.37 (satisfactory range). The mean difference was 4.43, which was statistically significant (*P* < 0.0001). In all clinical cases, post-training scores significantly exceeded pre-training values (*P* < 0.0001). Analysis of the CRI-HT-S scale showed the highest frequency in the domains of generally the criteria are met and approximately half of the criteria are met, demonstrating the effectiveness of the training.

**Conclusion:**

The online flipped classroom offers preliminary evidence as a feasible and valuable framework for developing clinical reasoning during clerkship. These findings provide a proof-of-concept for integrating blended learning into emergency medicine curricula to enhance diagnostic skills in high-acuity clinical domains.

**Supplementary Information:**

The online version contains supplementary material available at 10.1186/s12909-026-09094-x.

## Introduction

Clinical reasoning (CR) is a core competency in medical education, essential for developing accurate diagnostic hypotheses and ensuring high-quality patient care [1]. This complex process involves collecting and analyzing patient data, evaluating diagnostic options, and applying critical thinking skills to make informed clinical decisions [2, 3]. Developing proficiency in CR requires a robust knowledge base and the formation of structured “illness scripts” [4]. Despite its significance, CR is often taught informally during clinical rotations, leading to uncertain educational outcomes [1, 5]. Consequently, there is an urgent and sustained need to move beyond informal instruction and effectively integrate CR into medical curricula through innovative, structured pedagogical methods [6, 7].

Simultaneously, medical education continues to be shaped by digital technology, as “digital natives” increasingly expect technology-enhanced learning (TEL) to be integrated into their curriculum [8]. TEL facilitates learning through digital platforms and online content delivery, which has been shown to improve student engagement, motivation, and knowledge retention [9–12]. The exponential growth of virtual learning platforms and social media has made it easier for learners to navigate and integrate into the digital world, ultimately aiming for improved patient outcomes [13, 14].

The transition to these online pedagogical models was profoundly accelerated by the COVID-19 pandemic, which created major obstacles to traditional bedside teaching, such as clinical access restrictions, physical distancing requirements, safety concerns, and communication barriers imposed by personal protective equipment Crucially, the lessons learned from this period—specifically the ongoing need for flexible, standardized, and asynchronous strategies like the online flipped classroom—have persisted in the post-pandemic era [15, 16].

Despite these trends, limited research has been conducted on how to effectively use these technologies specifically for bedside instruction [17–19]. For decades, bedside teaching has helped students practice history-taking and examination skills under faculty mentorship [20]. However, several challenges arise in this setting, including faculty time constraints, high patient turnover, privacy concerns, and the inability to standardize clinical exposure [20, 21]. The lack of explicit attention to clinical reasoning as the core learning competency can further exacerbate these issues [22, 23].

The flipped classroom model offers a constructivist approach that stimulates higher-order cognitive skills and allows students to better apply their knowledge to patient care [24, 25]. This model enhances learner satisfaction by enabling self-pacing and reducing cognitive load through tailored pre-class assignments [26, 27]. By integrating these technologies into bedside instruction, educators can address traditional teaching challenges more optimally. Therefore, this study aimed to evaluate whether an online flipped classroom approach improves clinical reasoning performance, as measured by OSCE results, among medical students during their emergency medicine clerkship.

## Methods

### Design and ethical consideration

A pre-test–post-test quasi-experimental design was employed. This study was approved by the Research Ethics Committee of the Smart University of Medical Sciences (Code number: IR.VUMS.REC.1401.019). All participants provided written informed consent prior to their enrollment, and the study was conducted according to ethical principles of confidentiality and minimizing harm. Informed consent was obtained from all participants in written form.

### Participant and sampling

This study involved medical students enrolled in 2022–2023 at Jundishapur University of Medical Sciences in Iran, during the clerkship phase in the emergency department. The sample size was calculated using G Power software version 3.0.10. The maximum sample size was calculated based on the study by Kargol et al. Assuming a one-sided α = 0.05, power = 95%, effect size dz = 0.5 the computed sample size was 45 medical students.

All enrolled students completed both pre- and post-intervention assessments, and no missing data were observed.

### Inclusion and exclusion criteria

The inclusion criteria required participants to be officially enrolled in the 8-week Emergency Medicine clerkship, have successfully completed all pre-clinical core curriculum requirements, and possess a smartphone with the WhatsApp application to engage with the asynchronous electronic content, including video lectures and clinical cases. Furthermore, all participants were required to provide written informed consent prior to their enrollment in the study.

Regarding the exclusion criteria, students were removed from the final analysis if they had any prior formal exposure to flipped classroom models or clinical reasoning workshops to avoid prior-knowledge bias. Additionally, students who demonstrated low engagement—defined as missing more than 20% of the online pre-class activities or more than one bedside “think-aloud” teaching session—were excluded. Finally, the study excluded participants who failed to complete both the pre-test and post-test OSCE assessments and those who exercised their right to voluntarily withdraw from the study at any stage of the intervention.

### Educational design and implementation

**Fig. 1:**
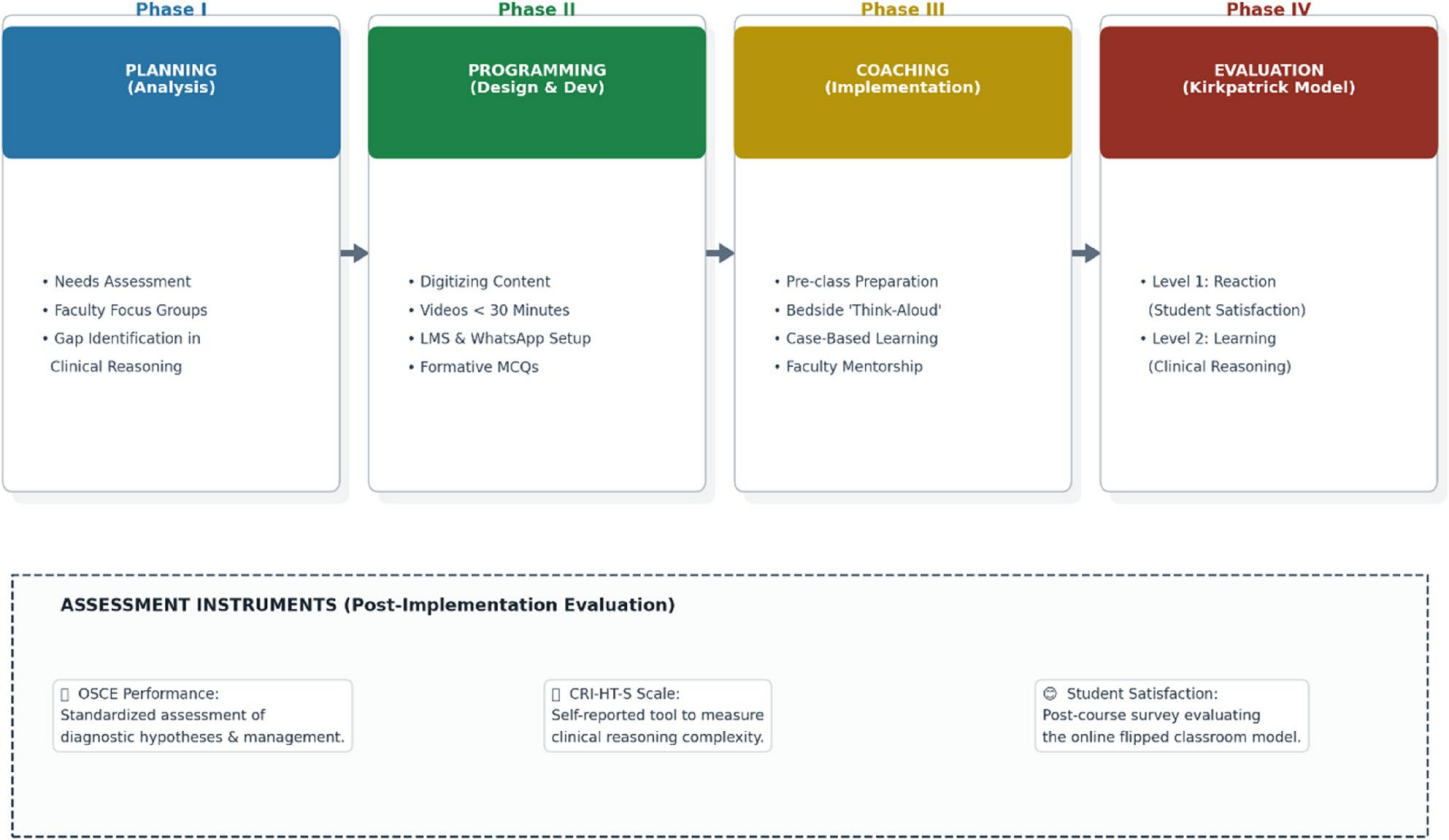
The modified ADDIE instructional design framework for the online flipped classroom, integrated with Kirkpatrick’s evaluation levels

Instructional Design Model The design of this online flipped classroom course was grounded in a modified version of the ADDIE instructional design model, specifically adapted by Eltahir [28] for virtual learning environments in higher education. This systematic framework facilitated the structured development of the clinical reasoning curriculum through four integrated stages: Planning, Programming, Coaching, and Evaluation (Fig. 1).

### Phase 1: Planning and needs assessment

In the initial planning stage, a comprehensive analysis of medical students’ characteristics and the learning context was conducted. We utilized a virtual focus group to gather data on age, gender, learning preferences, and prior knowledge. Final training needs were identified and prioritized through interviews with emergency medicine specialists and the application of the nominal group method to ensure the curriculum addressed the most critical clinical reasoning gaps.

### Phase 2: Programming and content development

During the programming phase, specific learning outcomes were defined, and corresponding educational materials were developed. These included narrated and annotated PowerPoint presentations and clinical case scenarios. Regarding the delivery of educational content, we selected a hybrid approach utilizing both a dedicated Learning Management System (LMS) and the WhatsApp platform. The rationale for incorporating WhatsApp was its high accessibility, user-friendly interface, and the ability to provide instant notifications, which ensured that students could engage with the materials regardless of their location or internet bandwidth constraints. This platform facilitated high interactivity, allowing for real-time peer-to-peer discussions and rapid clarification of queries. While the LMS served as the primary repository for structured modules, WhatsApp functioned as a dynamic tool for sharing short, video-based learning materials and clinical case scenarios, thereby fostering a continuous and mobile learning environment throughout the 8-week clerkship.

To ensure students had engaged with the asynchronous content before the synchronous sessions, a formative test was administered via the Learning Management System (LMS) following the viewing of each video module. This formative assessment consisted of 5 to 10 multiple-choice questions (MCQs) focusing on the key diagnostic and management principles covered in the video lectures. The test was designed to provide immediate feedback to the students, allowing them to identify gaps in their understanding before the bedside clinical reasoning sessions. Participation in these tests was mandatory, and the results were used by the instructors to tailor the subsequent ‘think-aloud’ sessions toward topics where students demonstrated lower proficiency.

### Phase 3: Implementation and Coaching (Online & Bedside)

#### The implementation followed a three-step flipped classroom process

Asynchronous Pre-class Phase: Students accessed electronic content via WhatsApp and the LMS. These materials consisted of short instructional videos (each less than 30 min) covering core emergency topics (e.g., trauma, shock, dyspnea, and pediatric fever). Students were required to review these videos and complete pre-class assignments and formative tests before the clinical sessions.

##### Synchronous phase

Synchronous 60-minute small-group, case-based discussions were conducted via Zoom, during which students verbalized their diagnostic reasoning and management priorities while faculty provided targeted feedback.

During the think-aloud process, students were required to explicitly verbalize their diagnostic reasoning, clinical hypotheses, and management priorities, while faculty facilitators used targeted probing questions to challenge assumptions and provide immediate corrective feedback.

Each 60-minute session followed a structured format: 10 min for case presentation, 20 min for student-led differential diagnosis and reasoning, 20 min for supervised think-aloud discussion and feedback, and 10 min for summary and key take-home messages.

The asynchronous video content focused on core theoretical concepts, diagnostic frameworks, and standardized management algorithms, whereas in-person bedside sessions were exclusively dedicated to the application of these concepts through real patient cases, prioritization of differential diagnoses, and justification of clinical decisions.

##### Collaborative learning

The “Coaching” element was integrated throughout, where instructors managed virtual collaborative activities and online case discussions to facilitate interactive learning and viewpoint exchange. Formative assessment was integrated throughout the intervention. Following the online modules, students completed short interactive quizzes via WhatsApp to ensure prerequisite knowledge. During bedside teaching, the ‘think-aloud’ technique served as a continuous assessment tool, where faculty provided immediate feedback on the students’ diagnostic logic and clinical reasoning pathways.

Phase 4: Evaluation: The effectiveness of the program was measured using the first and second levels of Kirkpatrick’s evaluation model. This included assessing student satisfaction (Reaction) and the impact on clinical reasoning skills through OSCE assessments (Learning).

In the traditional model, clinical reasoning was learned implicitly through observation and ad hoc questioning during bedside rounds, without structured case discussions, formal formative assessments, or explicit articulation of reasoning processes.

### Measurement instruments

Medical students’ clinical reasoning skills were assessed by two OSCEs, accepted objective measures completed at the end of their clerkship. The first OSCEs were administered to students before they completed their online flipped classroom training. Following completion of the online flipped classroom education, a second exam was administered.

A similar structure, resources, and assessment scale were utilized in both OSCEs. OSCE stations lasted 15 min. At each station, we assessed the clinical reasoning skills of medical students in emergency medicine. This was in areas such as trauma, shock, dizziness, headaches, dyspnea, chest pain, abdominal pain, and fever. A rest and question station were also provided. It is critical to note that each station’s case material was originally developed by emergency medicine faculty members. It has since been internally validated by experts to ensure accuracy and standardization.

The CRI-HT-S, or Clinical Reasoning Indicators History Taking Scale, was developed as an assessment tool for assessing medical students’ clinical reasoning skills in OSCE exams. This scale was originally developed by Furstenberg et al. and used for the assessment of clinical reasoning indicators during history taking. It is scored on a 5-point Likert scale, with 1 being the least aligned to the criteria and 5 being the most aligned. The Persian version of the CRI-HT-S scale was used in this study to assess participants’ clinical reasoning. A translation and back translation of the original questionnaire has been undertaken along with cross-cultural adaptation.

In order to ensure the validity of the questionnaire, ten medical education specialists assessed its content and face validity. The relevance, clarity, and structure of the questionnaires were evaluated. According to the study results, the questionnaire was appropriate for this study. Using Cronbach’s alpha coefficient, we evaluated its reliability. Based on the results, the scale had a high level of reliability, with an alpha coefficient of 0.91. As part of the OSCE exam, demographic data about the participants was collected, including their age, gender, marital status, and past experience with the exam.

To assess students’ satisfaction with our online flipped classroom clinical reasoning training course, we developed a questionnaire consisting of 17 questions. Ten medical educators were asked to complete the questionnaire to assess its content and face validity. Experts confirmed that the tool had both content and face validity. The questionnaire’s Cronbach’s alpha coefficient was also calculated to measure its reliability, and Cronbach’s alpha coefficient was 0.956. At the end of the course, a questionnaire was distributed to medical students and collected anonymously.

### Statistical analysis

Statistical analysis was conducted using SPSS software version 25. Qualitative variables were reported as numbers and percentages, while quantitative variables were expressed as mean and standard deviation. The Shapiro-Wilk test was performed to verify the normality of the data distribution. To compare the clinical reasoning scores before and after the intervention, paired t-tests were utilized. Furthermore, to determine the magnitude of the intervention’s impact, the effect size was calculated using Cohen’s d coefficient, where values of 0.2, 0.5, and 0.8 represented small, medium, and large effects, respectively [29].

### Demographic characteristics

A total of 45 medical students participated in this study. The gender distribution consisted of 27 (60.00%) females and 18 (40.00%) males. Regarding marital status, 27 (60.00%) students were single, while 18 (40.00%) were married. The mean age of the participants was 23.89 years (SD = 1.91), with ages ranging from 22.00 to 25.00 years. As summarized in Table 1, none of the participants had prior formal experience with flipped classroom models.

**Table 1:** Demographic characteristics of the medical students (*n* = 45)

Variable	Category	Frequency (*n*)	Percentage (%)
Gender	Male	18	40
	Female	27	60
Marital Status	Single	27	60
	Married	18	40
Age (years)	Mean (SD)	23.89 (1.91)	
	Range (Min–Max)	22.00–25.00	
Prior FC Experience	No	45	100

### Clinical Reasoning Performance

The implementation of the virtual flipped classroom significantly improved clinical reasoning competencies across all domains (*P* < 0.001). As detailed in Table 2, the total mean score increased from 25.97 (SD = 2.40) to 30.41 (SD = 3.37), reflecting a robust educational gain with a large effect size (Cohen’s d = 1.51). The mean difference of 4.44 (95% CI: 3.94–4.92) highlights a consistent upward trend in student performance. Specifically, the most substantial quantitative improvement was observed in the “Headache” domain (Mean Diff = 3.07). Beyond raw scores, the Clinical Reasoning Index (CRI) demonstrated a critical qualitative shift, rising from a baseline of 3.25 to 3.79. This evolution is visually summarized in Fig. 1, which illustrates that all clinical scenarios successfully surpassed the professional proficiency threshold of 3.0.

**Table 2:** Comparison of Clinical Reasoning Scores and Clinical Reasoning Index (CRI) Achievement

Clinical Domains	Pre Mean	Post Mean	Mean Diff.	95% CI (Lower–Upper)	*P*-value	Pre CRI	Post CRI	Cohen’s d
Trauma	26.33	29.16	2.83	1.11–4.54	< 0.001	3.11	3.64	0.96
Shock	27.97	30.58	2.61	2.01–3.19	< 0.001	3.27	3.82	1.05
Dizziness	28.02	30.56	2.54	2.05–3.02	< 0.001	3.28	3.82	1.12
Headache	27.82	30.89	3.07	2.52–3.60	< 0.001	3.38	3.82	1.18
Dyspnea	28.75	30.09	1.34	1.18–2.14	< 0.001	3.09	3.76	0.62
Chest Pain	28.08	30.22	2.14	1.52–2.75	< 0.001	3.22	3.78	0.88
Abdominal Pain	28.44	31.2	2.76	2.12–3.39	< 0.001	3.29	3.9	1.04
Pediatric Fever	28.46	30.56	2.1	1.44–2.73	< 0.001	3.34	3.82	0.91
Total Score	25.97	30.41	4.44	3.94–4.92	< 0.001	3.25	3.79	1.51

*P-values corrected for multiple comparisons using Bonferroni adjustment

**Fig. 2:**
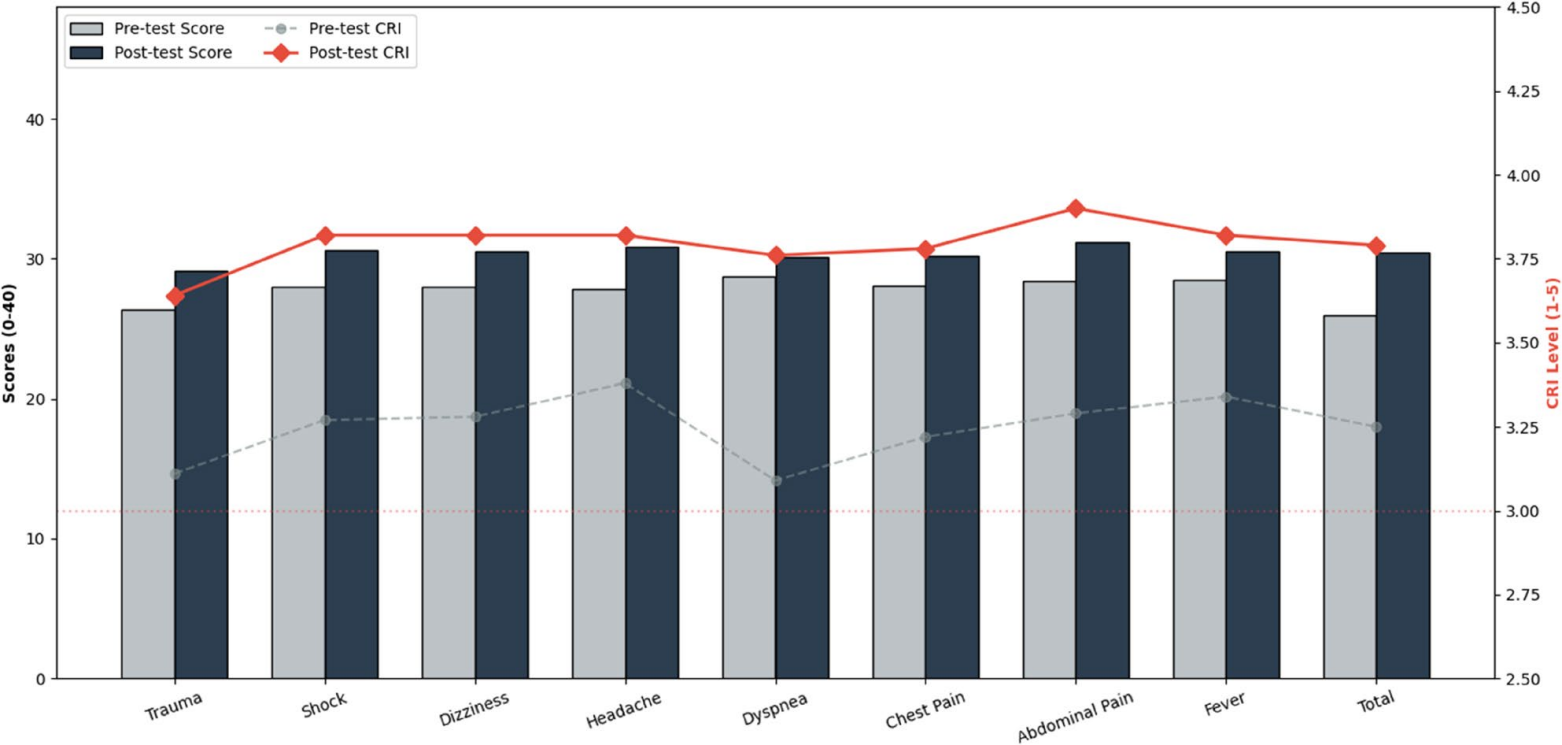
Frequency distribution of achievement levels across clinical reasoning criteria post-intervention

### Distribution of clinical reasoning achievement levels

Analysis of the frequency distribution across eight clinical reasoning criteria (Table 3) showed a predominant shift toward higher proficiency. Post-intervention, student responses were primarily concentrated in the “Generally met” (Point 4) and “In complete compliance” (Point 5) categories, particularly in domains such as Trauma, Shock, and Abdominal Pain.

**Table 3:** Frequency distribution of student responses across clinical reasoning criteria and achievement levels (CRI-HT-S) for eight emergency clinical scenarios

**Clinical reasoning criteria**	**Criteria not met** **Point = 1**								**Is relatively non-compliant with the criteria** **Point = 2**								**Approximately half of the criteria are met** **Point = 3**			
	**Trauma**	**Shock**	**Dizziness**	**Headache**	**Dyspnea**	**Chest Pain**	**Abdominal Pain**	**Fever**	**Trauma**	**Shock**	**Dizziness**	**Headache**	**Dyspnea**	**Chest Pain**	**Abdominal Pain**	**Fever**	**Trauma**	**Shock**	**Dizziness**	**Headache**
Discussion initiating and guiding	4	2	0	2	3	1	2	1	8	5	3	6	1	7	2	6	6	8	6	9
Being able to recognize relevant information and respond appropriately to it	0	0	0	1	0	1	2	0	9	2	6	3	4	3	2	2	10	12	7	6
A diagnosis of the symptoms is made	1	0	0	1	2	0	0	1	3	2	2	3	1	5	2	3	12	11	12	12
Inquiring about pathological symptoms in a specific manner	0	3	0	0	2	3	1	0	7	4	5	2	4	5	2	2	9	8	10	10
Organize the questions logically	0	2	2	0	1	3	1	1	9	7	5	5	9	2	4	1	8	9	10	11
Discuss the information obtained with the patient	3	2	5	0	3	3	4	4	4	6	3	6	3	5	2	5	8	10	9	8
Creating abstracts	3	1	5	1	1	5	3	4	4	6	4	6	6	5	4	2	2	8	8	7
Gathering information and evaluating the effectiveness of the conversation.	1	0	1	1	1	0	1	0	3	1	2	4	1	0	3	5	14	6	8	7

**Clinical reasoning criteria**	**Approximately half of the criteria are met** **Point = 3**				**Generally, the criteria are met** **Point = 4**								**In complete compliance with the criteria** **Point = 5**							
	**Dyspnea**	**Chest Pain**	**Abdominal Pain**	**Fever**	**Trauma**	**Shock**	**Dizziness**	**Headache**	**Dyspnea**	**Chest Pain**	**Abdominal Pain**	**Fever**	**Trauma**	**Shock**	**Dizziness**	**Headache**	**Dyspnea**	**Chest Pain**	**Abdominal Pain**	**Fever**
Discussion initiating and guiding	7	11	9	6	22	21	23	18	19	12	18	18	5	9	13	10	15	14	14	14
Being able to recognize relevant information and respond appropriately to it	8	9	2	4	13	15	21	17	24	17	21	10	13	16	11	18	9	15	10	17
A diagnosis of the symptoms is made	13	13	11	10	21	22	21	19	14	12	17	18	8	10	10	10	15	15	11	13
Inquiring about pathological symptoms in a specific manner	14	12	14	14	21	20	17	15	17	11	17	18	8	10	13	18	18	14	18	11
Organize the questions logically	9	13	12	12	13	17	16	21	12	13	20	20	15	10	12	8	14	14	14	11
Discuss the information obtained with the patient	12	12	5	10	22	11	16	15	12	12	15	10	8	16	12	16	14	13	15	16
Creating abstracts	8	4	4	11	2	20	15	21	13	19	20	13	13	10	13	10	10	12	14	15
Gathering information and evaluating the effectiveness of the conversation.	14	4	6	12	13	14	14	14	13	17	17	13	14	24	20	19	16	24	18	15

As shown in Fig. 2, superior performance was particularly evident in domains such as Trauma, Shock, and Abdominal Pain. Specifically, students excelled in “Gathering information” and “Recognizing relevant information,” with a substantial majority achieving top-tier scores. Conversely, the “Criteria not met” (Point 1) category saw minimal frequency, indicating that the online flipped classroom model successfully aligned students’ reasoning skills with professional clinical standards.


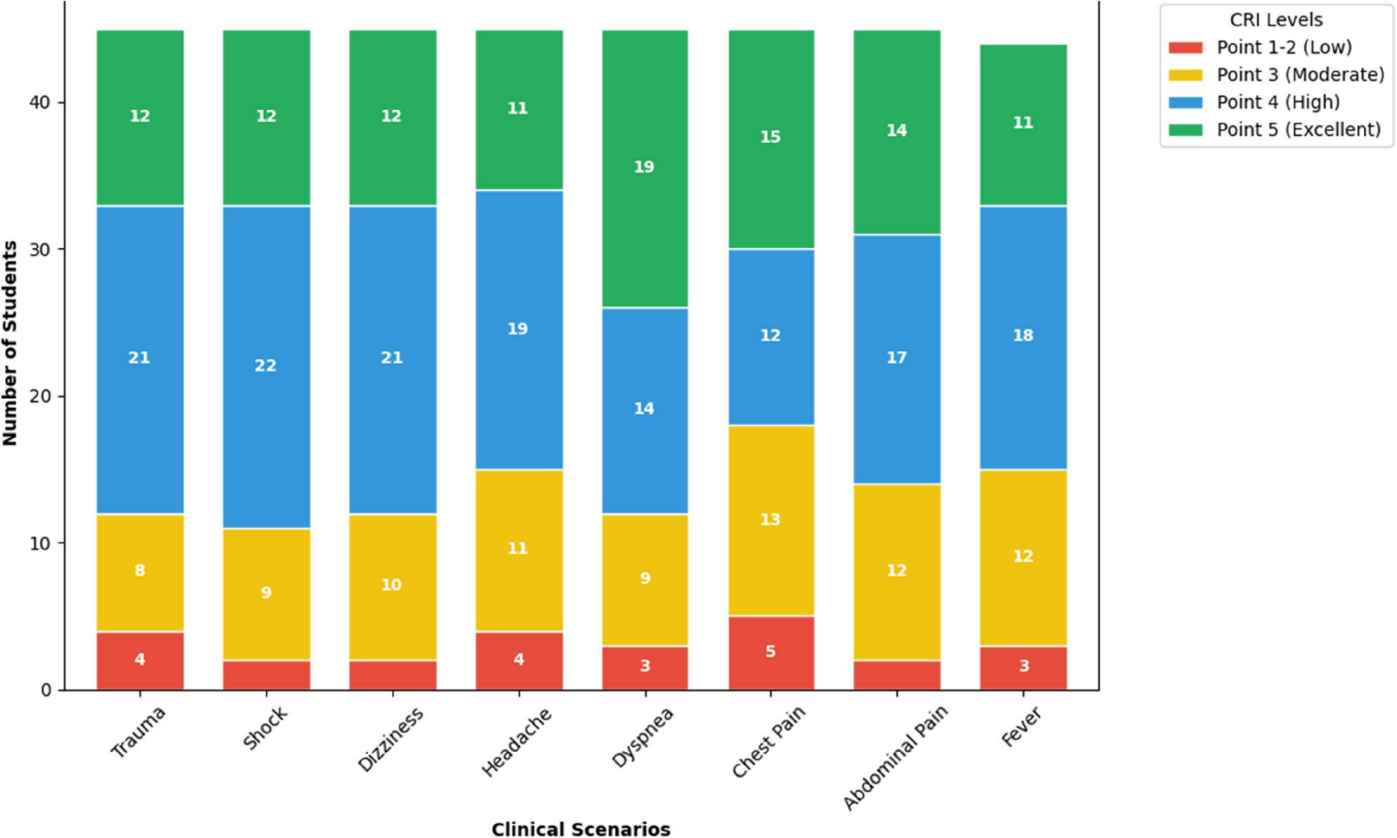


### Course evaluation and student satisfaction

The effectiveness of the virtual flipped classroom course was further evaluated through student satisfaction surveys. The descriptive analysis revealed a high level of satisfaction among participants, with an overall mean score of 3.47 (SD = 0.71) on a 5-point Likert scale.

**Fig. 3:**
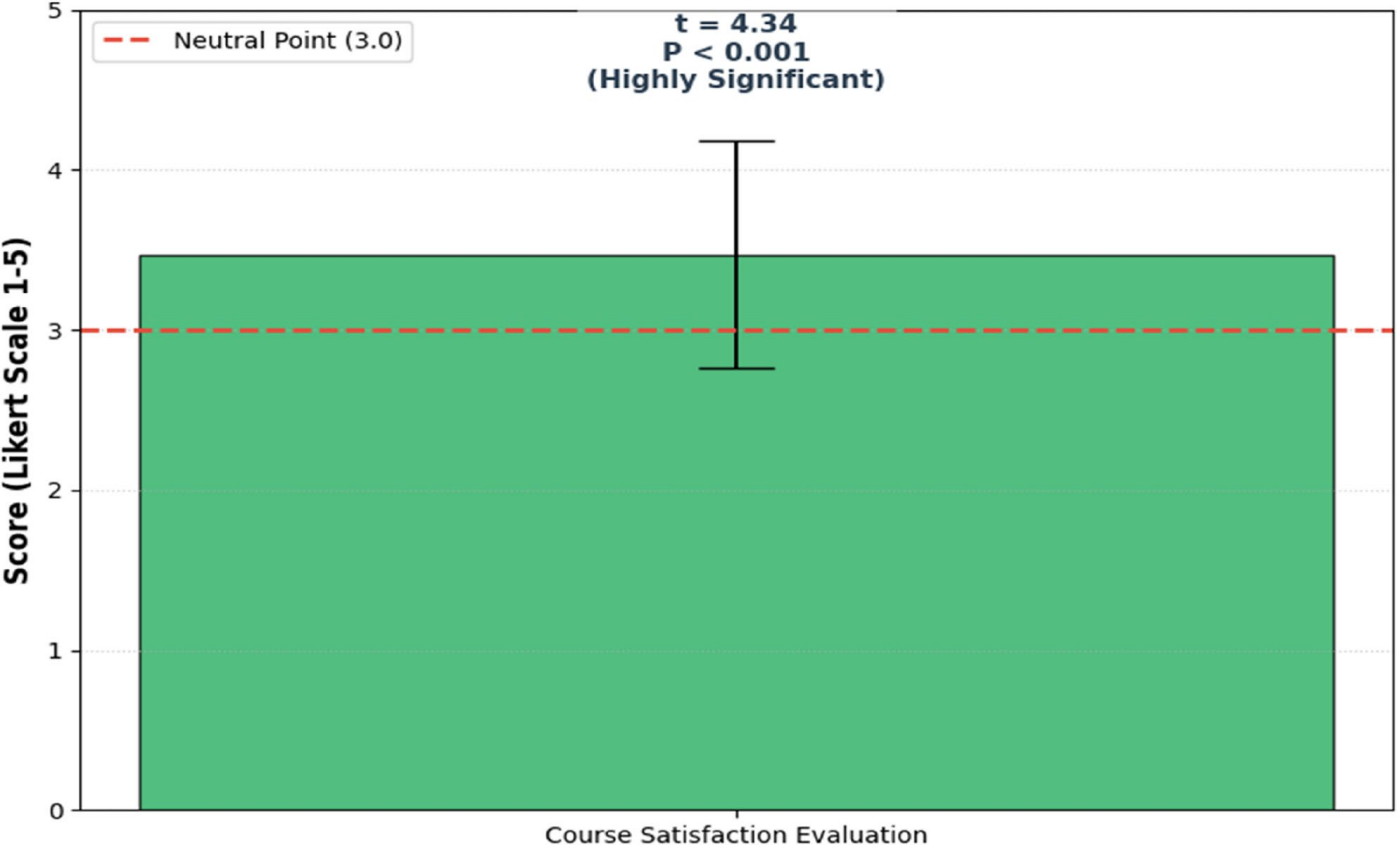
Statistical evaluation of student satisfaction with the online flipped classroom training course

As illustrated in Fig. 3, a one-sample t-test was conducted to compare the satisfaction mean against the theoretical neutral point of 3.0. The results indicated a statistically significant positive perception of the training course (t = 4.34, df = 44, *P* < 0.001). The mean difference of 0.47 (95% CI: 0.27–0.68) confirms that the intervention successfully met the students’ educational expectations. Furthermore, the qualitative distribution showed that the highest frequencies of responses were centered on the criteria being “generally” or “completely” met, reinforcing the pedagogical value of the online flipped model in clinical reasoning instruction.

The bar represents the mean satisfaction score (3.47 ± 0.71 SD) on a 5-point Likert scale. The dashed red line indicates the theoretical neutral point (3.0). Results from the one-sample t-test (t = 4.34, df = 44, *P* < 0.001) demonstrate a significantly positive perception of the course effectiveness among medical students.

## Discussion

The global evolution of medical curricula necessitates a transition from passive didacticism to robust, active learning frameworks that mirror the complexity of modern clinical practice. The current study demonstrates that the online flipped classroom (OFC) model functions as a high-impact pedagogical intervention, significantly augmenting both the quantitative scores and the qualitative depth of clinical reasoning among medical students. This improvement suggests that the OFC model does not merely facilitate knowledge acquisition but effectively reshapes the cognitive architecture required for diagnostic inquiry in high-acuity environments [24, 30]. By empowering students to transition from passive recipients to self-directed problem-solvers, this study highlights how active engagement facilitates the mastery of higher-order cognitive tasks.

A critical finding of this study is the intervention’s capacity to bridge the historical “theory-practice gap” [31] within bedside education. Traditional clinical instruction is often fragmented by the unpredictable nature of patient presentations; however, the OFC provides a standardized, high-fidelity simulation of emergency scenarios [32]. By leveraging OSCEs—which serve as a surrogate for real-world clinical competence [33]—the current study observed a significant elevation in the Clinical Reasoning Index (CRI), ensuring students surpassed professional proficiency thresholds.

Notably, we observed nuanced differences in performance across clinical domains, likely attributed to the intrinsic complexity of the subjects. High-acuity domains such as Trauma and Shock demonstrated the most robust gains, as their management follows structured, life-saving protocols (e.g., ABCDE approach) [34] that align perfectly with the pedagogical “scaffolding” provided by the flipped classroom [35]. This suggests the model is particularly effective for subjects with high cognitive load, where pre-class preparation allows students to internalize pathophysiology before applying it in high-pressure scenarios.

Beyond general performance gains, the current study aligns with the findings of Paul et al., who demonstrated that flipped instruction is specifically superior for augmenting clinical skills during clerkships compared to traditional or purely online formats [36]. This evidence further supports the efficacy of flipped classrooms as integrated teaching and assessment strategies that yield significant results in clinical skills training [37–39]. Furthermore, some studies suggest that flipped models are generally more effective than traditional methods for teaching reasoning [25, 40], often resulting in high student satisfaction [41, 42].

These outcomes reinforce the hypothesis that combining case-based learning with flipped instruction creates a synergistic effect [43, 44]. As suggested by Zhang et al. [45] and Diel et al. [46], such a framework allows students to practice critical problem-solving in a risk-free environment, potentially mitigating clinical errors and enhancing patient safety during real-world bedside encounters. This mastery is further supported by Tsao et al., who reported superior outcomes for evidence-based medicine competencies—specifically the “Ask, Acquire, Appraise, and Apply” categories—compared to traditional methods [47].

Furthermore, the shift towards digital and blended frameworks in medical curricula is increasingly supported by recent literature. A 2025 study by Sayed et al. highlighted that undergraduate medical students value structured and interactive environments for clinical reasoning learning [1]. While their research primarily focused on student perceptions, our study provides the objective empirical evidence that such interactive online frameworks directly translate into improved clinical performance. This confirms that the online flipped classroom not only meets student pedagogical expectations but also effectively enhances the diagnostic logic required in high-acuity emergency settings.

Beyond quantitative gains, faculty observations and student feedback in our study revealed that the OFC model served as a ‘psychological scaffold,’ significantly increasing learner confidence and reducing anxiety during high-acuity encounters. Faculty noted that students demonstrated a more proactive approach to bedside problem-solving, suggesting that the model effectively translates theoretical knowledge into perceived clinical self-efficacy.

This study has several methodological limitations inherent to its design and the clerkship setting. First, the quasi-experimental, single-group pre–post design and the small number of students per rotation limit the ability to attribute observed improvements solely to the online flipped classroom intervention. Natural maturation, concurrent clinical exposure, and test-retest effects may also have contributed to performance gains. Second, the lack of a formal longitudinal follow-up precludes assessment of long-term retention of clinical reasoning skills and potential impact on patient care outcomes. Third, while we included a brief descriptive summary of student and faculty experiential feedback, no formal qualitative or mixed-methods analysis was conducted, limiting insights into perceptions and implementation challenges. Fourth, although multiple comparisons were performed across clinical domains, we applied Bonferroni correction and calculated effect sizes to mitigate Type I error. Finally, the study’s results reflect a pilot/feasibility evaluation; definitive conclusions regarding effectiveness, generalizability, and scalability require future controlled trials (e.g., RCTs) with larger samples across multiple sites.

## Conclusion

In conclusion, this study suggests that an online flipped classroom approach integrated into emergency medicine clerkship is a feasible and acceptable educational strategy for supporting clinical reasoning development. The observed improvements in OSCE performance provide preliminary evidence of potential benefit; however, due to the quasi-experimental design, lack of a control group, and absence of formal qualitative evaluation, these findings cannot be interpreted as definitive evidence of effectiveness or superiority over traditional instructional methods. As a pilot, hypothesis-generating study, this work provides initial insights that can inform future controlled and mixed-methods research, including qualitative investigations to capture student and faculty experiences, identify implementation challenges, and better understand how technology-enhanced strategies may facilitate clinical reasoning development in emergency medicine training.

## Supplementary Information

Supplementary Material 1.

Supplementary Material 2.

## Data Availability

The datasets used during the current study are available from the corresponding author on reasonable request.
