# Supplementary material for: Enhancing diagnostic logic in high-acuity care: evidence from an online flipped classroom intervention in emergency medicine
Source: BMC Med Educ. 2026 Apr 9;26:822. doi: 10.1186/s12909-026-09094-x (PMC13217878; doi:10.1186/s12909-026-09094-x)
Supplement: Supplementary file 1 — Supplementary Material 1. [file 12909_2026_9094_MOESM1_ESM.pdf]

Virtual University of Medical Sciences

Research Ethics Committees Certificate

<table>
<tr><td>Approval ID:</td><td>IR.VUMS.REC.1401.019</td><td rowspan="2">Approval Date:</td><td rowspan="2">2022-10-31</td></tr>
<tr><td>Evaluated by:</td><td>Research Ethics Committees of Virtual University of Medical Sciences</td></tr>
<tr><td>Status:</td><td colspan="3">Approved</td></tr>
<tr><td>Approval Statement:</td><td colspan="3">The project was found to be in accordance to the ethical principles and the national norms and standards for conducting Medical Research in Iran.
Notice:
1. Although the proposal has been approved by the Biomedical Research Ethics Committee, meeting the professional and legal requirements is the sole responsibility of the PI and other project collaborators.
2. This certificate is reliant on the proposal/documents received by this committee on 2022-10-31. The committee must be notified by the PI as soon as the proposal/documents are modified.</td></tr>
<tr><td>Thesis Title:</td><td colspan="3">Design, implementation and evaluation of an flipped clinical reasoning course in a virtual context for medical students in Ahvaz Jundishapur University of Medical Sciences</td></tr>
<tr><td>Supervisor:</td><td colspan="3">Name: nooshin kohan
Email: nu.kohan@gmail.com
Name: mehdi sayyah
Email: sayahbargard@gmail.com</td></tr>
<tr><td>Student:</td><td colspan="3">Name: Ali Delirrooyfard
Email: adelir2891@gmail.com</td></tr>
</table>

A. Khojasteh

Dr. Arash Khojasteh
Committee Director
Virtual University of Medical Sciences

Dr. Babak Sabet Divshali
Committee Secretary
Virtual University of Medical Sciences
