# Supplementary material for: Enhancing diagnostic logic in high-acuity care: evidence from an online flipped classroom intervention in emergency medicine
Source: BMC Med Educ. 2026 Apr 9;26:822. doi: 10.1186/s12909-026-09094-x (PMC13217878; doi:10.1186/s12909-026-09094-x)
Supplement: Supplementary file 2 — Supplementary Material 2. [file 12909_2026_9094_MOESM2_ESM.docx]

**Clinical Reasoning Indicators – History Taking (CRI-HT)**

Clinical reasoning indicators | 1 | 2 | 3 | 4 | 5 |

|  |  |  |  |  |  |
| --- | --- | --- | --- | --- | --- |

1. Taking the lead in the conversation

The student takes control of the interview in order to get the required information.

| O | O | O | O | O |

2. Recognizing and responding to relevant information

The student shows that s/he recognizes relevant information by e.g. responding with obvious interest to them. | O | O | O | O | O |

3. Specifying symptoms

The student makes targeted inquiries to capture the symptoms in more detail which s/he considers to be important. | O | O | O | O | O |

4. Asking specific questions that point to pathophysiological thinking

The student’s questions indicate that s/he is considering specific causes for certain symptoms.

| O | O | O | O | O |

5. Putting questions in a logical order

The student asks the questions in a logical order and not according to a list. | O | O | O | O | O |

6. Checking with the patient

The student assures her-/himself by checking with the patient that her/his clinical thinking is based on correct information. | O | O | O | O | O |

7. Summarizing

The student summarizes her/his collected information aloud as soon as they have reached a meaningful level. | O | O | O | O | O |

8. Collected data and effectiveness of the conversation

The student collects sufficient, high quality data at reasonable speed. | O | O | O | O | O

**Scoring Legend:**

(1) Does not meet the criterion

(2) Does rather not meet the criterion

(3) Partly meets the criterion

(4) Rather meets the criterion

(5) Fully meets the criterion
